# The focus of attention is similar to other memory systems rather than uniquely different

**DOI:** 10.3389/fnhum.2014.00056

**Published:** 2014-02-10

**Authors:** Olivia Beaudry, Ian Neath, Aimée M. Surprenant, Gerald Tehan

**Affiliations:** ^1^École de Psychologie, Université de MonctonMoncton, NB, Canada; ^2^Department of Psychology, Memorial University of NewfoundlandSt. John’s, NL, Canada; ^3^Department of Psychology, University of Southern QueenslandSpringfield, QLD, Australia

**Keywords:** focus of attention, proactive interference, working memory, memory systems, embedded processes model

## Abstract

According to some current theories, the focus of attention (FOA), part of working memory, represents items in a privileged state that is more accessible than items stored in other memory systems. One line of evidence supporting the distinction between the FOA and other memory systems is the finding that items in the FOA are immune to proactive interference (when something learned earlier impairs the ability to remember something learned more recently). The FOA, then, is held to be unique: it is the only memory system that is not susceptible to proactive interference. We review the literature used to support this claim, and although there are many studies in which proactive interference was not observed, we found more studies in which it was observed. We conclude that the FOA is not immune to proactive interference: items in the FOA are susceptible to proactive interference just like items in every other memory system. And, just as in all other memory systems, it is how the items are represented and processed that plays a critical role in determining whether proactive interference will be observed.

When short-term memory was created in late 1950s/early 1960s, it ushered in a view in which memory is divided into multiple memory systems, each of which has different properties, each of which operates according to different rules and principles, and each of which is responsible for storing different types of information and supporting memory in different types of situations. This quickly became the dominant view of the field. An alternate view emphasizes processing rather systems, and suggests that memory performance is dependent on the relation between the processing done at encoding and the processing done at retrieval rather than on which store holds the information. The debate over systems vs. processes was perhaps most prominent at the turn of the century (see, for example, the volume edited by Foster and Jelicic, 1999) but it is fair to say that the debate is still ongoing (see Surprenant and Neath, 2009).

One commonality between these two approaches is that they emphasize differences (either in store or in processing): Differences in memory performance are taken as indicating a different underlying memory system or different process. Memory, whether seen as a system or process, is chopped up, divided, subdivided, and partitioned into ever decreasing portions.

An alternate view emphasizes similarities rather than differences by advocating the search for general principles of memory (Surprenant and Neath, 2009). In contrast to both the systems and processes view, this functional approach focuses on larger and larger portions of the memory puzzle. It is this view that provided the impetus for the current paper, and while our question is narrow, its implications are broad and the answer relevant to theorists of any persuasion: Is the focus of attention (FOA), unlike all other proposed memory systems, immune to proactive interference?

## Proactive interference (PI)

Proactive interference (PI) is the finding that old information can interfere with the ability to remember new information (for a review, see Neath and Surprenant, in press). It is observed in all memory systems, including the perceptual representation system, procedural memory, semantic memory, long-term episodic memory, and short-term or working memory. Perhaps the most well-known example was reported by Underwood (1957). He pointed out that in most memory experiments, subjects receive multiple lists, and the researcher plots performance averaged over all of the trials. In contrast, he re-plotted the data as a function of the number of previous lists and found that the more prior lists, the worse the level of recall. He and others (for a review, see Crowder, 1976) suggested that PI was a major source of forgetting in memory.

According to criteria proposed by Surprenant and Neath (2009), PI is a candidate for a principle of memory. First, it is an empirical regularity, being observable in all memory systems; the only system that is claimed to be immune from PI is the FOA. Second, it can serve as an intermediate explanation or as a more abstract statement about a set of results. The results from a variety of studies can be explained by appealing to PI as the reason for worse performance in one condition compared to an appropriate control, and this can link otherwise disparate findings. Third, it offers useful information about how memory works. One example relevant to many people is remembering the password for a new account rather than a password for a previously created account.

Being an “intermediate explanation” is not sufficient, however. PI itself still needs to be explained. Most theoretical accounts of PI are similar to the basic description: earlier items interfere with memory for later items (e.g., Henson, 1998; Mensink and Raaijmakers, 1988). The differences between the various accounts have to do mostly with the specific nature of the effect of the prior item. For example, Watkins and Watkins (1975) proposed an explanation based on cue overload, the finding that the more items a cue subsumes, the less effective the cue will be. According to this account, the category (or other salient feature) of the to-be-remembered items can be a significant retrieval cue. It is as if the subject were asking at test, “What were the three letters I just saw?” As more trials occur, the number of potential responses increases. The cue, in this case, letters, becomes less and less helpful, as there are more and more letters to choose from. If the type of category changes, a new cue is used that subsumes far fewer items and thus performance improves. A different type of account was offered by Baddeley (1976), who framed PI in terms of distinctiveness. The idea is easy to appreciate through an analogy with visual perception: If you are looking at a set of evenly spaced telephone poles, the closer poles are easy to distinguish from one another, but as the poles get further away from the viewer, the poles start to blend together. PI occurs when the “poles” appear close together and PI can be minimized or eliminated if the experiment is altered such that the “poles” are spaced further apart. This idea has been developed and expanded by Brown et al. (2007). Note that in both cases, a critically important component is how the to-be-remembered items are represented.

One reason there is not a universal theoretical account of PI may be because it is multiply determined. For example, consider the difference between a recall study and a recognition study. In the former, the subject receives a short list of items that are all of the same type (for example, numbers), and is then asked to recall them. This repeats for a number of lists, and the typical result is that recall decreases over trials, indicating the build up of PI. At some point, the type of item changes (for example, to consonants) and performance on the first changed trial improves, indicating the release from PI. Now, consider a recognition task. Again, a short list of items that are all of the same type is presented. Instead of recalling the items, though, the subject is presented with a probe item and is asked to judge if the probe was on the list. The probe could be on the list; it could be a new, never-before seen item; or it could be an item from a previous list. As reviewed in more detail below, probes that were in previous lists typically result in slower response times compared to novel probes. This is also a from of PI—an earlier item is interfering with memory for a later item—but this may have a different fundamental cause than the PI observed in the recall case. For example, the former may be due to cue overload whereas the latter may be due to a conflict between two processes, one based on familiarity and one based on recollection. It is possible that these are different effects, in the sense of different fundamental causes. However, our claim is not that there is only one kind of PI, but rather, effects of PI can be observed in all memory systems, including the Focus of Attention (FOA).

## The focus of attention

A number of researchers have proposed that some types of memory, all of which resemble William James’ primary memory, are immune to PI (e.g., Wickens et al., 1981; Halford et al., 1988; Schweickert, 1993), with the most recent example being Cowan’s (1999) embedded processes model. This model distinguishes between (1) long-term memory; (2) working memory, which is considered to be the activated portion of long-term memory; and (3) the FOA. The model conceives of working memory as a set of processes carried out in long-term memory, and that working memory is simply the subset of information in long-term memory that is temporarily activated. This is in direct contrast to other conceptions of working memory, such as Baddeley’s (2003) version, in which working memory is seen as fundamentally separate from long-term memory.

The FOA is immune to PI because of the nature of the representational state. Items stored in the FOA “are, in a sense, already retrieved; they reside in a limited-capacity store, eliminating the retrieval step in which PI arises” (Cowan, 2001, p. 103). This same idea is invoked in other accounts, even if the term FOA is not used. For example, Schweickert (1993) posits “direct readout” and Wickens et al. (1981) explicitly invoke the Jamesian notion that some items were never “cut off in consciousness”. All these conceptions, whether termed FOA or not, posit a limited capacity of approximately four items (e.g., Wickens et al., 1981; Halford et al., 1988; Cowan, 2001). One reason for this limit is that, as documented below, these researchers point to a number of studies in which PI is readily observed with lists with more than four items but is not observed when the lists had four items or fewer.

Other researchers have proposed that the FOA has a capacity of only a single item (e.g., Garavan, 1998; McElree, 2001). One possible reason for the differing estimates may be the use of different paradigms, but a review of these differing capacity estimation procedures is beyond the scope of the current paper.^1^ Nonetheless, we do note that there are studies that demonstrate PI when only a single item is thought to be held in the FOA (e.g., Ruusuvirta et al., 2008).

Instead, we focus our attention on the most common paradigm used to support the claim of immunity to PI, a variation of the Sternberg (1966) memory scanning task. In addition, we include only those studies in which only four or fewer items are required to be stored in the FOA.

Our approach is based on the following. Currently, many theorists propose that the FOA is immune to PI. A similar claim used to be made about implicit memory. In particular, Graf and Schacter (1987) compared implicit (incidental learning and indirect test) and explicit (intentional learning and direct test) memory for word pairs. They found PI for the explicit task but no evidence of PI for the implicit task. A subsequent study by Lustig and Hasher (2001), however, used a slightly modified procedure and did observe PI. The key difference is that in Lustig and Hasher’s (2001) design, the interfering information was relevant at the time of test given the processing that the subjects are asked to do; in the Graf and Schacter (1987) study, the interfering item was not relevant. (The experimental details are described at length in Neath and Surprenant, 2003, pp. 147–150). Our hypothesis is that it is possible the same situation that existed in the implicit memory literature exists in the FOA literature. That is, in the studies that show immunity to PI, the interfering item is not relevant to the task, and therefore does not cause interference. According to this explanation, in studies where the interfering item is relevant, there should be interference.

## Evidence suggesting immunity to proactive interference (PI)

Halford et al. (1988) used a modified Sternberg (1966) memory scanning task. In Experiment 1, lists of 4 or 10 words were presented simultaneously on a computer screen. One second after the list disappeared, a probe appeared. Subjects were asked to indicate if they had seen the probe item within the memory set by pressing the “yes” or “no” buttons. PI was manipulated by presenting words from the same taxonomic category on three consecutive trials and words from another category for the next three consecutive trials, and so on. The first trial of a category was considered to be a low PI trial because the three previous trials had stimuli from a different category. The third trial of a category was considered to be a high PI trial because it had words from the same category as the two preceding trials. When 10 items were shown, response times were faster and performance was more accurate on the low PI trials than on high PI trials. Having prior lists with the same category interfered with performance on later trials, a classic example of PI. However, there was no difference between the low PI and high PI trials when only four items were shown. According to the embedded processes model, when items are in the FOA, PI is not observed. Because four unrelated items can be stored within the FOA, the PI manipulation does not have an effect and performance does not differ between the low and high PI trials. In contrast, 10 items greatly exceeds the capacity of the FOA, so other memory systems are recruited; it is these other systems that produce PI.

Experiment 2 replicated these results, with a few changes. Halford et al. (1988) employed lists of 4, 6, 8, or 10 words in the memory set, and in addition, rhyming categories were used instead of semantic categories. Once again, PI was observed when the lists had six or more items, but not when the list had only four items.

Our review of the literature found a number of studies that replicate this result using the same basic paradigm: Presence of PI when the memory set contains more than four items and an absence of PI when the number of to-be-remembered items is 4 or fewer (Wickens et al., 1981, 1985; Cowan et al., 2005; Cowan and Saults, 2013). All of these studies used a procedure similar to that used by Halford et al. (1988), in that taxonomic category was manipulated to induce PI. Thus, there is considerable evidence supporting the view that the FOA is immune to PI.

## Evidence suggesting susceptibility to proactive interference (PI)

Finding immunity to PI when the to-be-remembered items are thought to be stored in the FOA is informative only to the extent that there exist no studies that do observe PI. We reviewed the literature and found more studies that *did* observe PI than studies that *did not* observe PI (see Table 1). Again, we limit the review to those studies using a Sternberg memory scanning task with four or fewer items, although there are many studies in other paradigms with similar results. We briefly review these studies to give a suggestion of why the authors of these studies observed PI whereas those noted above did not. We then discuss possible causes for the difference in the results.

**Table 1:** **Studies using a modified Sternberg task that found items in the focus of attention are (1) immune to PI (left column) or (2) susceptible to PI (right column)**.

FOA is Immune to PI	FOA is Susceptible to PI
Cowan and Saults (2013)	Atkins et al. (2011)
Cowan et al. (2005)	Atkins et al. (1974)
Halford et al. (1988)	Brannelly et al. (1989)
Humphreys and Tehan (1992)	Carroll et al. (2010)
Sanders and Willemsen (1978)	Craig et al. (2013)
Wickens et al. (1981, 1985)	Hanley and Scheirer (1975)
	Jonides et al. (1998, 2000)
	McElree and Dosher (1989)
	Monsell (1978)

Hanley and Scheirer (1975) used a modified Sternberg task but rather than words used letter pairs as the stimuli. On each trial, subjects saw a total of three items presented sequentially followed by a probe item. For four trials in a row, the subjects saw letter pairs, and on the fifth trial the stimuli was switched to two-digit numbers. After four consecutive trials with numbers, they were switched back to letters. Thus, Trial 1 was a low PI trial and Trial 4 was a high PI trial. Response times were faster on Trial 1 than Trial 4, suggesting that PI built up over trials. This was true for both positive probes (the probe item was in the memory set) and negative probes (the probe item was not in the memory set).

Carroll et al. (2010) reported what is essentially a replication of Hanley and Scheirer (1975). The main changes were including nonwords instead of letter pairs in Experiment 1 and symbols (such as *!@#$&*) instead of letter pairs in Experiment 2. The rationale was that the three letter pairs might be represented as six items in the FOA, thus exceeding its capacity. Three one-syllable pronounceable nonwords (e.g., *dess, mout, swip*) would not exceed the capacity. In both experiments, response time increased from Trial 1 to Trial 4, suggesting that items from the earlier trials interfered with memory on the later trials. In addition, release from PI was observed in that response times decreased with a change of materials. This was the case for both positive and negative probes.

Atkinson et al. (1974) showed subjects 2, 3, 4, or 5 words, followed by a probe. The probe could be one of the items in the list (a P or positive trial), or it could be a word that was not on the list (an N or negative trial). Atkinson et al. (1974) manipulated the number of times the probe word had been experienced. N1 meant that the word was being seen for the first time. N2 meant that the word was being seen for the second time, having also been seen as a distractor on the immediately preceding trial. N3 meant that the word was being seen for the third time, having been seen on the preceding list as both a member of the list and as the probe. Mean RT systematically increased for correct responses on N1, N2, and N3 trials. Importantly, even when there were only two items in the memory set, the mean RT for an N1 trial was 634 ms compared to 658 and 668 ms on N2 and N3 trials respectively. The same was true for trials on which there were three items in the memory set: RTs increased from 661 to 679 to 714 ms. Thus, PI was directly related to how many times a negative probe had been previously experienced.

Monsell (1978) used lists of 1, 2, 3 or 4 consonants. On each trial, the consonants were presented sequentially for 2 s each. The probe appeared immediately after the final item (no delay condition) or after 500 ms (delay condition). In the latter condition, a vowel was presented during the delay period and subjects were asked to name the vowel. As in other studies, subjects were asked to press one of two buttons to indicate whether the probe was on the list. For half of the trials, positive probes were used (i.e., they were in the set) and for the remaining trials, a negative probe was used (i.e., the probe was not in the set). There were two types of negative probes: Half of the negative probes consisted of recent probes. These had appeared as to-be-remembered stimuli on the previous trial but had not been presented in the memory set on the current trial. The remaining half had novel probes, which had not been presented recently. Subjects were slower and less accurate for recent probes than novel probes even when the list contained as few as two items. It was only for the 1-item lists that there was no difference between novel and recent probes. PI, then, was observed on lists with 2, 3, and 4 items.

McElree and Dosher (1989) also observed PI in short lists when using negative probes. In Experiment 2, lists of 3, 4, 5, or 6 two-syllable nouns were presented sequentially on the computer screen. After the presentation of the last word, the probe word appeared. Response times were slower for both distant and recent negative probes compared to positive probes. In addition, RTs were on the order of 50 ms slower for recent negative probes compared to distant negative proves. Once again, PI was observed with lists with as few as three items.

Jonides et al. (1998) presented four letters on each trial followed by a probe. Recent probes had appeared in the previous trial, whereas novel probes had not appeared in the two preceding trials. Subjects gave slower and less accurate responses for recent negative probes compared to novel probes.

Jonides et al. (2000) conducted a similar study but included older adults (aged between 61 and 72) in addition to college-aged subjects. PI due to recent negative probes was again observed, with the effect even larger for the older compared to younger subjects.

Atkins et al. (2011) used four words on each trial followed by a probe. Again, they examined both recent and novel positive and negative probes. Subjects were slower to correctly respond to recent negative probes than non-recent negative probes. There was also a higher false alarm rate for recent negative probes than non-recent negative probes. Atkins et al. (2011) also included a condition with concurrent articulation and found even larger effects of PI than in the quiet condition.

In a second experiment, Atkins et al. (2011) included a manipulation of semantic information. Memory sets were comprised of items from two semantic categories (fruits and countries) but still contained four items. In the categorized condition, memory sets were comprised of words within the same category (e.g., all fruit words). The category label of the probe item at test either matched or mismatched the category presented in the memory set. When it was mismatched, the correct response was a negative response. There was also a mixed condition, a mixture of fruits and country words were presented within the memory set which makes categorization of memory sets difficult. For the mismatch trials, PI was reduced compared to the match negative trials, but even so, effects of PI were still observed on the mismatch negative trials.

Craig et al. (2013) reported 5 experiments. The basic paradigm was to present four items simultaneously followed by a probe. One type of probe indicated to subjects that they should respond whether the probe was one of the four items. Other types of probes, manipulated over the experiments, indicated to subjects that they should make a category membership judgment or a perceptual judgment (i.e., was the probe presented in italics?). In general, effects of PI were observed on the memory task but not on the semantic or perceptual judgment task.

There are a number of other studies we could cite, but each has a characteristic that may make it less clear as an example of PI in the FOA. For example, Brannelly et al. (1989) used lists of two or four items, but included a filled delay. PI was observed even for the 2-item list, but a proponent of the FOA account might argue that such a delay means that some system other than the FOA was responsible for the outcome.

As a second example, we briefly mention a related paradigm. Tehan and colleagues (e.g., Tehan and Humphreys, 1995, 1996; Tolan and Tehan, 1999; Humphreys and Tehan, 1999; Ralph et al., 2011) have often used a two list paradigm. On some proportion of trials, a list of four items is presented and subjects are asked about that list. It could be via a probe, via serial recall, or via cued recall. The data of most interest, however, come from the other trials. Here, the first list is presented, and then a signal is shown indicating that list is not being tested. A second list is shown, and subjects are asked about the second list. The items in the preceding untested list can be designed to cause PI. Using this procedure, Tehan and colleagues have repeatedly demonstrated PI when the interfering items in list 1 are relevant given the task required for list 2. For example, Tehan and Humphreys (1996), Experiment 2) demonstrated interference by semantic content with cued recall. The probe question might ask the subject to recall the item from the immediately preceding list that is a vegetable and in both the control and interference conditions the list might contain the word *cabbage*. The key manipulation is whether the immediately preceding list contained *carrot* (same category) or *pumpkin* (different category). Responses were less accurate in the same than different category conditions indicating PI. Using this type of paradigm, Tehan and colleagues have shown PI caused by a number of different dimensions. What is most important from this series of studies is that PI is not always observed: It is observed only when the dimension that caused PI was one that was relevant at test given the type of memory task.

## Discussion

Within the embedded processes model (Cowan, 1999, 2001), the FOA is viewed as a memory store that is immune to PI. The reason is because of the representational state of the items: The items are thought to be present in mind, and “are, in a sense, already retrieved” and therefore, no retrieval from other memory systems is needed (Cowan, 2001, p. 103). Within this framework, PI is seen as occurring during the retrieval process but because retrieval is not needed for items in the FOA, those items cannot be affected by PI. Other theorists have proposed similar ideas (e.g., Wickens et al., 1981; Halford et al., 1988; Schweickert, 1993). When the number of items in the list is small (usually around 4 or fewer), the items are said to be represented in a state that allows direct readout or are in a state in which the items have never left consciousness. When more items are in the list, then retrieval has to occur, and then PI can occur. Initial studies using a modified Sternberg task seemed to support these views: Trials with four or fewer items showed no effects of PI whereas trials with more than four items did show PI. However, all of these studies induced PI by semantic or taxonomic information.

In contrast, there is a larger set of otherwise identical studies in which PI is observed with four or fewer items. The key difference is that the majority of these studies did not use semantic or taxonomic information to induce PI. For example, studies in which the type of item periodically changes (e.g., from numbers to letters) show the build-up and release of PI. As another example, some studies manipulate whether the probe item has been seen in previous trials and find that recently seen probes cause interference. In our view, the key difference between those studies showing PI and those which do not is whether the potentially interfering item is relevant given the memory task. In those few studies where semantic information does cause PI, it is because the task has been slightly changed to make the semantic dimension relevant.

If one assumes that memory is primarily a discrimination task (e.g., Neath and Brown, 2006; Brown et al., 2007), then the pattern of results is readily explainable. Items can be represented in memory as points in multidimensional space; which particular dimensions are relevant depends on the task and how the items are processed. Similarly, the ability to discriminate between those items that were on the target list from competitor items also depends on the relative differences between the items on those dimensions.

Consider first the study by Hanley and Scheirer (1975). On the first trial, numbers are shown. The test is easy, because only 4 numbers have been seen so far, despite the fact that numbers have relatively few relevant dimensions on which they differ from each other. On the second trial, the test is harder because the numbers from Trial 1 are now competitors when trying to remember the numbers from Trial 2. On Trials 3 and 4, even more competitors are present, leading to more PI. On Trial 5, however, the stimulus class is changed from numbers to letters, so the number of competitors is reduced and release from PI is seen. If one agrees with the idea of cognitive economy—that people tend to use processes that minimize effort and resources—then the results from studies like those reported by Halford et al. (1988) are readily understood without needing to invoke the unique property of immunity to PI. Instead, one assumes that subjects use “shallow” processing for short lists because performance is good enough.^2^ One consequence is that the dimension that is supposed to cause interference—taxonomic category—is just not relevant to this type of processing. It is only when the task becomes sufficiently hard that the more economical processing begins to falter that “deeper” processing is performed and now the taxonomic dimension becomes relevant.

In some studies, the interfering item is made relevant by reusing items from previous lists as probes for the current list. “Deep” processing is not necessary here, but interference will occur because the probe is now a plausible competitor. Finally, in studies such as Tehan and Humphreys (1995), the taxonomic dimension is made relevant by changing the question: Recall the vegetable. Now, interference based on semantic processing occurs, even though there are as few as four items. It is the task at test that makes the potentially interfering dimension relevant.

We noted above that our review is limited to those studies where the number of items is at or below the capacity of the FOA, and those that use the paradigm most often claimed to demonstrate immunity. One consequence is that all studies considered used verbal stimuli. During the review process, one reviewer noted that the FOA might be immune to PI when non-verbal stimuli are used in a different paradigm. As an example, the reviewer cited Lin and Luck (2012), who found no evidence of PI in a standard change detection task that used simple features as the stimuli. A review of the visual working memory literature is beyond the scope of the current article, but we note two responses.

First, it is important to keep in mind that we fully expect there to be a large number of paradigms in which PI will not be observed. Our view does not predict that PI must always be observed or must be observed in every possible task; rather, it predicts that PI must be observable in all memory systems. Just as one can construct a task that taps long-term episodic memory that does not yield PI, or a task that taps short-term episodic memory that does not yield PI, or a task that taps implicit memory that does not yield PI, or a task that taps semantic memory that does not yield PI, or a task that taps procedural memory that does not yield PI, one can also construct a task that taps the FOA that does not yield PI (see Neath and Surprenant, in press, for examples of PI in all of the aforementioned memory systems). The existence of such failures to observe PI does not compromise the logic of our paper: The claim made by a number of researchers (e.g., Wickens et al., 1981; Halford et al., 1988; Cowan, 1999, 2001; Schweickert, 1993) is that the FOA is immune to PI. A single instance of demonstrating PI when the items are in the FOA is sufficient to disprove the claim.

Second, one of the goals of pursuing the principles agenda is to identify boundary conditions, and we acknowledge this may very well be one: With more complex visual (but non-verbal) stimuli, PI can and does occur, and with other paradigms, PI can and does occur. With very simple stimuli in one specific task, PI may not occur. The question then becomes the following: Is this boundary condition sufficient to invalidate the general principle? Note that this is a different claim from that which is the focus of this paper. A claim that certain tasks are immune from PI is quite different from one claiming a particular memory system (or subsystem) is immune to PI, and it is also a claim that is entirely compatible with the principles approach.

## Conclusions

There are no accounts of PI that require PI be always observed at all times in all tests of a particular memory system. Rather, the various accounts of PI predict that PI will be observed when, for example, a retrieval cue becomes overloaded (Watkins and Watkins, 1981) or an item becomes relatively less distinct than competitors (Baddeley, 1976; Brown et al., 2007). Consistent with these and other accounts of PI, our review of the relevant literature found some studies that found evidence of PI when items were presumably held in the FOA and some studies that did not find evidence of PI. This pattern of results is, we claim, the same as is observed for other memory systems. For example, there are a large number of studies that fail to find PI in long-term memory tasks. In the long-term memory literature, there are no researchers who posit that long-term memory is immune to PI on the basis of those studies that did not observe PI. Rather, it is simply a reflection of the fact that although a memory system might be susceptible to PI, it need not show PI in every single study.

In this respect, the FOA is just like every other memory system: It is susceptible to PI under the appropriate conditions. There are presumably many tasks in which items are thought to be in the FOA that do not show evidence of PI. But there are a sufficiently large number of studies that do demonstrate PI when then items are in the FOA. Thus, the claim that items in the FOA are immune to PI is disproven. Whether PI occurs depends on how the items are represented and on how the items are processed. If an item could potentially interfere with a target but the dimension that could lead to interference is not relevant to the task, then PI will not be observed. In contrast, if an item could potentially interfere with a target and the task is such that the dimension that could lead to interference is now relevant to the task, then PI will be observed. This applies whether the item is thought to be in implicit memory, procedural memory, episodic memory, long-term memory, short-term memory, working memory, or, as we have argued here, the FOA.

## Conflict of interest statement

The authors declare that the research was conducted in the absence of any commercial or financial relationships that could be construed as a potential conflict of interest.

## Footnotes

^1^Cowan (2011) argues that the two competing capacity claims are reconcilable; the interested reader is referred to that article for further details.

^2^We use the terms “shallow” and “deep” in a descriptive sense only; we are not invoking the full theoretical account proposed in the Levels Of Processing framework (Craik and Lockhart, 1972).
